# Inhibitory effects of isocryptotanshinone on gastric cancer

**DOI:** 10.1038/s41598-018-27638-0

**Published:** 2018-06-18

**Authors:** Zhang-Ming Chen, Lei Huang, Miao-Miao Li, Lei Meng, Song-Cheng Ying, A-Man Xu

**Affiliations:** 1 Department of Gastrointestinal Surgery, Department of General Surgery, First Affiliated Hospital of Anhui Medical University, Hefei, 230022 China; 2 Department of Immunology, School of Basic Medical Sciences, Anhui Medical University, Hefei, 230032 China

## Abstract

Gastric cancer (GC) is one of the most common digestive malignancies globally, and the prognosis of patients with advanced tumors remains poor. Isocryptotanshinone (ICTS), isolated from Salvia miltiorrhiza, was found to inhibit the proliferation of lung and breast cancer cells. However, whether ICTS has anticancer activities against GC is unknown. In the present study, we reported that the proliferation of GC cells was inhibited by ICTS in a dose- and time-dependent manner. After treatment with ICTS, GC cells were arrested in the G1/G0 phase of cell cycle and the apoptotic cells were induced in a dose-dependent manner. Additionally, ICTS suppressed the expression of cell cycle- and apoptosis-associated proteins (*e.g*., Cyclin D1, phosphorylated Rb, E2F1, Mcl-1, Bcl-2, and Survivin). ICTS inhibited the phosphorylation of STAT3 in a dose-dependent manner. Down-regulated STAT3 attenuated the expression of Cyclin D1, p-Rb, and Survivin, which remarkably increased the sensitivity of ICTS in GC cells; overexpression of STAT3 restored the cell growth and proliferation and the protein expression suppressed by ICTS. ICTS also suppressed the xenograft tumor growth in BALB/c nude mice. Together, these data indicate that ICTS inhibits GC proliferation by inducing G1/G0 cell cycle arrest and apoptosis via inhibiting the STAT3 signaling pathway.

## Introduction

Gastric cancer (GC) ranks as the 5^th^ most common cancer and the 2^nd^ leading cause of cancer-related death worldwide^1–3^. It is especially prevalent in China^4^. Radical resection of the whole tumor mass combined with D2 lymph node excision remains the only approach potentially offering chance of cure^5–7^. Adjuvant chemo(radio)therapy further improves the postoperative overall survival^8,9^. Over the past decades, although GC incidence and mortality have both declined thanks to remarkable advances in multi-disciplinary diagnosis and treatment modalities, the prognosis of patients with advanced-stage tumors remains very poor^10–14^. The exploration of more effective agents with fewer adverse events to complement conventional therapy is necessary to improve the outcome of GC patients^15–17^. The herbal therapeutics deriving from traditional Chinese medicine are promising for GC treatment^18,19^.

Salvia miltiorrhiza, also called “Danshen” in Chinese, is one of the most commonly-used traditional Chinese medicines. It has been applied clinically to treat hyperlipidemia, hepatic fibrosis, chronic renal failure, gynecological diseases, and ischemic diseases^20,21^. Recent studies have revealed that Danshen extracts also possess diverse properties (*e.g*., anti-inflammatory, antibacterial, anti-oxidative, and anticancer activities)^22,23^. Cryptotanshinone, one of major components extracted from Danshen, has been extensively studied worldwide as a novel antitumor drug^24,25^. Recently, isocryptotanshinone (ICTS), an analogue of cryptotanshinone, was shown to inhibit the proliferation of lung and breast cancer cells by inducing apoptosis and pro-death autophagy^26,27^. However, until now, the effect of ICTS on GC is obscure. In the present study, we for the first time revealed that ICTS suppressed the proliferation of human GC cell lines SGC-7901 and MKN-45 via inducing cell cycle arrest at the G1/G0 phase and apoptosis by inhibiting the STAT3 signaling pathway, suggesting ICTS as a potential therapeutic agent against GC.

## Results

### ICTS inhibited GC cell proliferation

**Figure 1:**
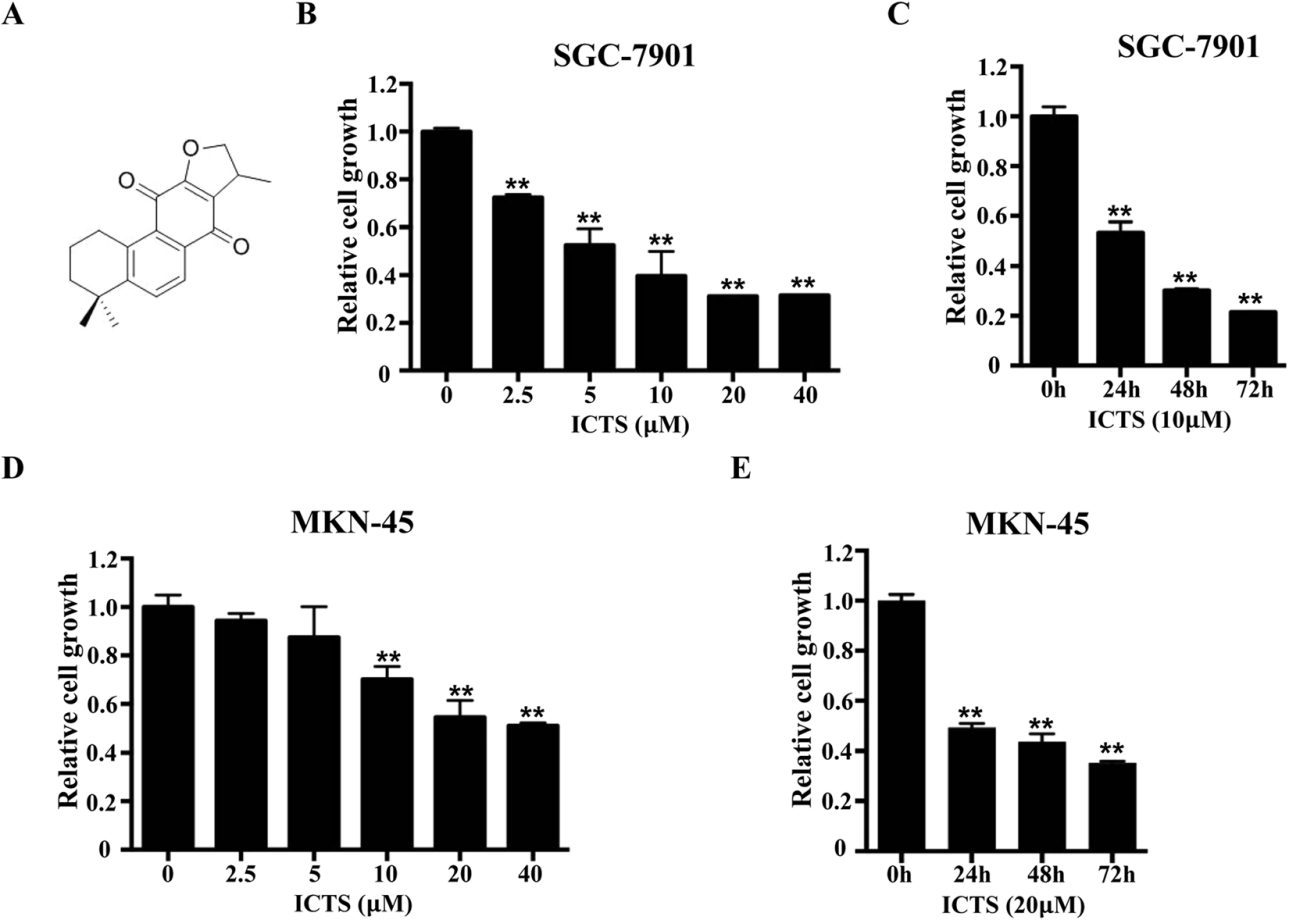
Effect of isocryptotanshinone on proliferation of SGC-7901 and MKN-45 cells. (**A**) shows the chemical structure of ICTS. SGC-7901 (**B**) and MKN-45 (**D**) cells were treated with the indicated concentration of ICTS for 48 hours, and the proliferation was assessed using the Cell Counting Kit (CCK)−8 assay. SGC-7901 (**C**) and MKN-45 (**E**) cells were treated with 10 or 20 *μ*M ICTS for the indicated time respectively, and the cell growth was determined using the CCK-8 assay. Data were expressed as mean ± standard error of triplicates from a representative experiment. *P < 0.05 and **P < 0.01 versus the control group (DMSO or 0 h group). ICTS, isocryptotanshinone.

To determine whether ICTS inhibits the proliferation of GC cells, we assessed the growth of SGC-7901 and MKN-45 cells after treatment with ICTS using the CCK-8 assay. The chemical structure of ICTS is shown in Fig. 1A. As shown in Fig. 1B, ICTS inhibited the proliferation of SGC-7901 cells in a dose-dependent manner, and the IC_50_ was 6.77 *μ*M. As shown in Fig. 1C, after treatment with 10 *μ*M ICTS, the proliferation of SGC-7901 cells was suppressed in a time-dependent manner. Additionally, ICTS induced inhibition of SGC-7901 cell proliferation mainly during the first 24 hours. Meanwhile, as shown in Fig. 1D and E, MKN-45 cell growth was also inhibited by ICTS in a dose- and time-dependent manner, which was consistent with the results in SGC-7901 cells, with an IC_50_ of 33.1 *μ*M. Interestingly, the inhibition of SGC-7901 cell proliferation induced by ICTS at lower concentration was more potent compared with that of MKN-45 cells. These results indicated that ICTS suppressed the proliferation of GC cells and might function as a GC suppressor.

### ICTS induced GC cell cycle arrest at the G1/G0 phase

**Figure 2:**
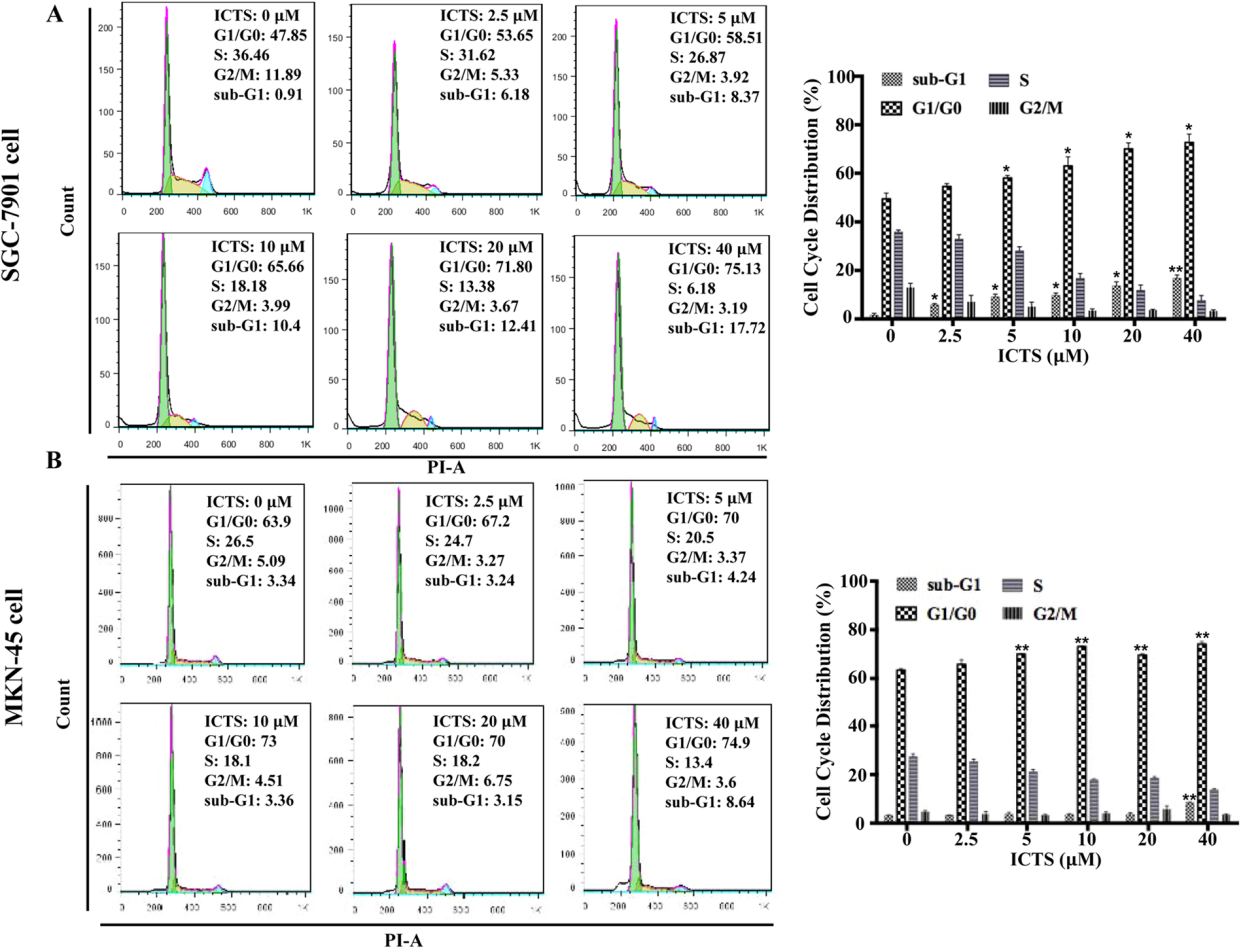
Effect of isocryptotanshinone on cell cycle in SGC-7901 (**A**) and MKN-45 (**B**) cells. Cells were serum-starved overnight and treated with the indicated concentration of ICTS and serum for 24 hours, and the contents of DNA stained by propidium iodide were detected using FACScan flow cytometry. The percentages of cells in the sub-G1, G1/G0, S, and G2/M phases were determined by the Flow Jo software. Data were expressed as mean ± standard error of triplicates from a representative experiment. *P < 0.05 and **P < 0.01 versus the control group (DMSO group). ICTS, isocryptotanshinone.

Cell proliferation is controlled by the progression of cell cycle. Here, we assessed whether ICTS inhibited cell proliferation via regulating cell cycle. As shown in Fig. 2A, treatment with ICTS for 24 hours arrested SGC-7901 cells in the G1/G0 phase of the cell cycle in a dose-dependent manner, which was consistent with the results shown in Fig. 1B. At a concentration of 10 *μ*M, ICTS markedly increased the proportion of SGC-7901 cells in the G1/G0 phase from 47.9% to 65.7%. Meanwhile, the increase of the SGC-7901 cell proportion in the G1/G0 phase was accompanied with a concomitant decreasing proportion of cells in the S and G2/M phases of the cell cycle. Additionally, treatment with 10 *μ*M ICTS increased the SGC-7901 cell number in the sub-G1 phase significantly, which suggested that ICTS might also play an essential role in the regulation of apoptosis. Similarly, as shown in Fig. 2B, ICTS also induced cell cycle arrest in the G1/G0 phase in MKN-45 cells and increased cell proportion in the sub-G1 phase of cell cycle at higher concentration. Together, the data showed that ICTS arrested GC cell cycle in the G1/G0 phase.

### ICTS induced GC cell apoptosis

**Figure 3:**
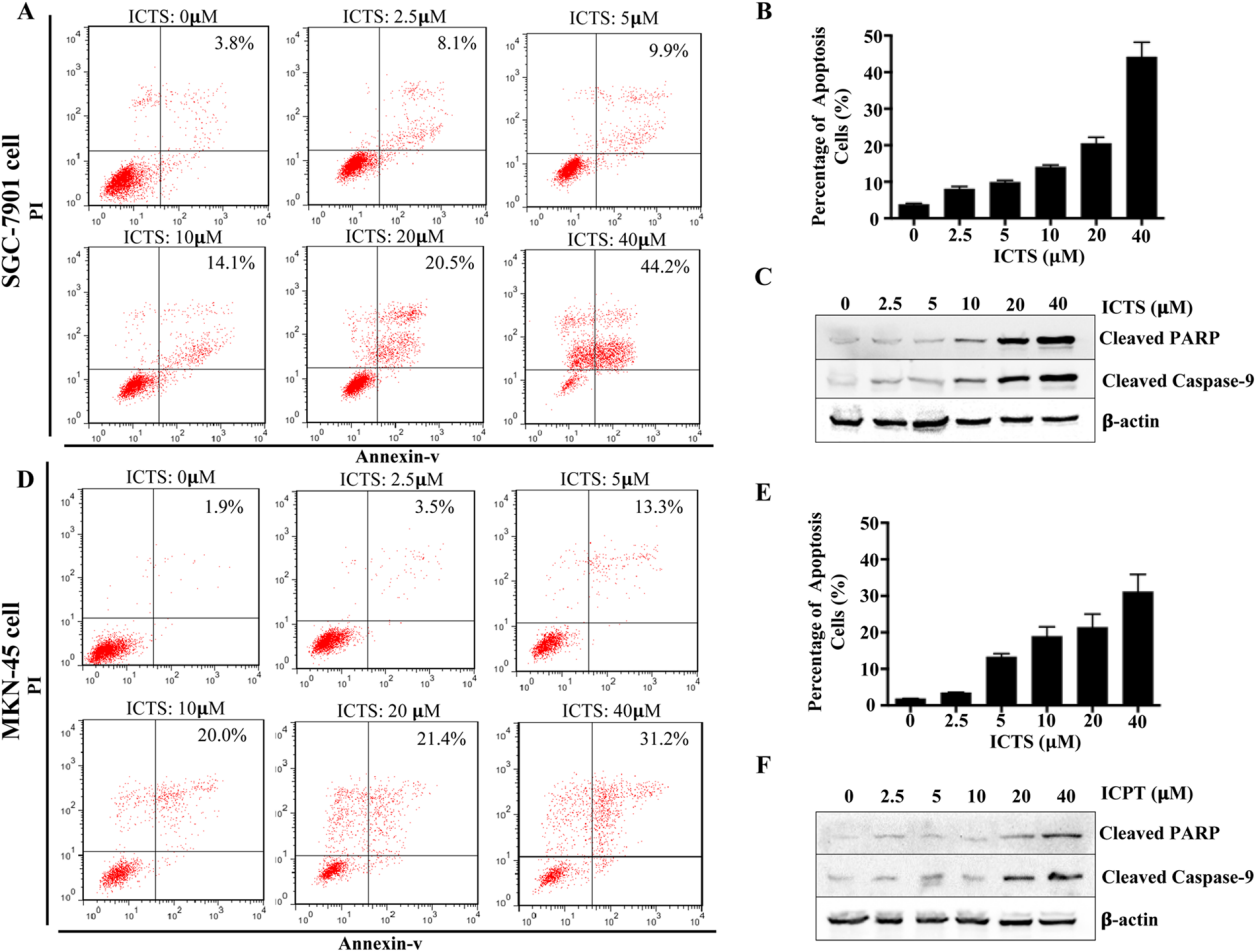
Effect of isocryptotanshinone on apoptosis of SGC-7901 and MKN-45 cells. Cells were exposed to the indicated concentration of ICTS for 24 hours, and the SGC-7901 (**A**) and MKN-45 (**D**) cells stained using propidium iodide and annexin-v were detected using FACScan flow cytometry. The percentages of apoptotic SGC-7901 (**B**) and MKN-45 (**E**) cells were determined by the Flow Jo software and outputted in histogram. Expression of cleaved PARP and cleaved caspase-9 in SGC-7901 (**C**) and MKN-45 (**F**) cells were determined by western blot. Data were expressed as mean ± standard error of triplicates of one representative experiment. β-actin was used as the loading control. ICTS, isocryptotanshinone.

To determine whether the ICTS-mediated growth inhibition in SGC-7901 and MKN-45 cells is associated with apoptosis, the apoptotic cells were further examined by the flow cytometry analysis. As shown in Fig. 3A and B, the percentage of Annexin^+^/PI^+^ SGC-7901 cells induced by ICTS after 24 hours was significantly increased from 3.8% to 44.2% in a concentration-dependent manner. The results also indicated that even low concentration of ICTS (*e.g*., 2.5 and 5 *μ*M) showed apoptosis-inducing effects and the apoptotic cell number was remarkably increased by ICTS at higher concentration (40 *μ*M) after 24 hours. Apoptosis, which is initiated by active caspase-9, induces cleaved-PARP expression and cell death^28^. As shown in Fig. 3C, the expression of cleaved caspase-9 and PARP were significantly upregulated in SGC-7901 cells after exposed to ICTS for 24 hours. As shown in Fig. 3D and E, ICTS also promoted MKN-45 cell apoptosis in a dose-dependent manner. Additionally, ICTS treatment increased the expression of cleaved caspase-9 and PARP remarkably in MKN-45 cells (Fig. 3F). Thus, these results showed that ICTS induced apoptosis in GC cells.

### ICTS regulated expression of cell cycle- and apoptosis-associated proteins in GC cells

**Figure 4:**
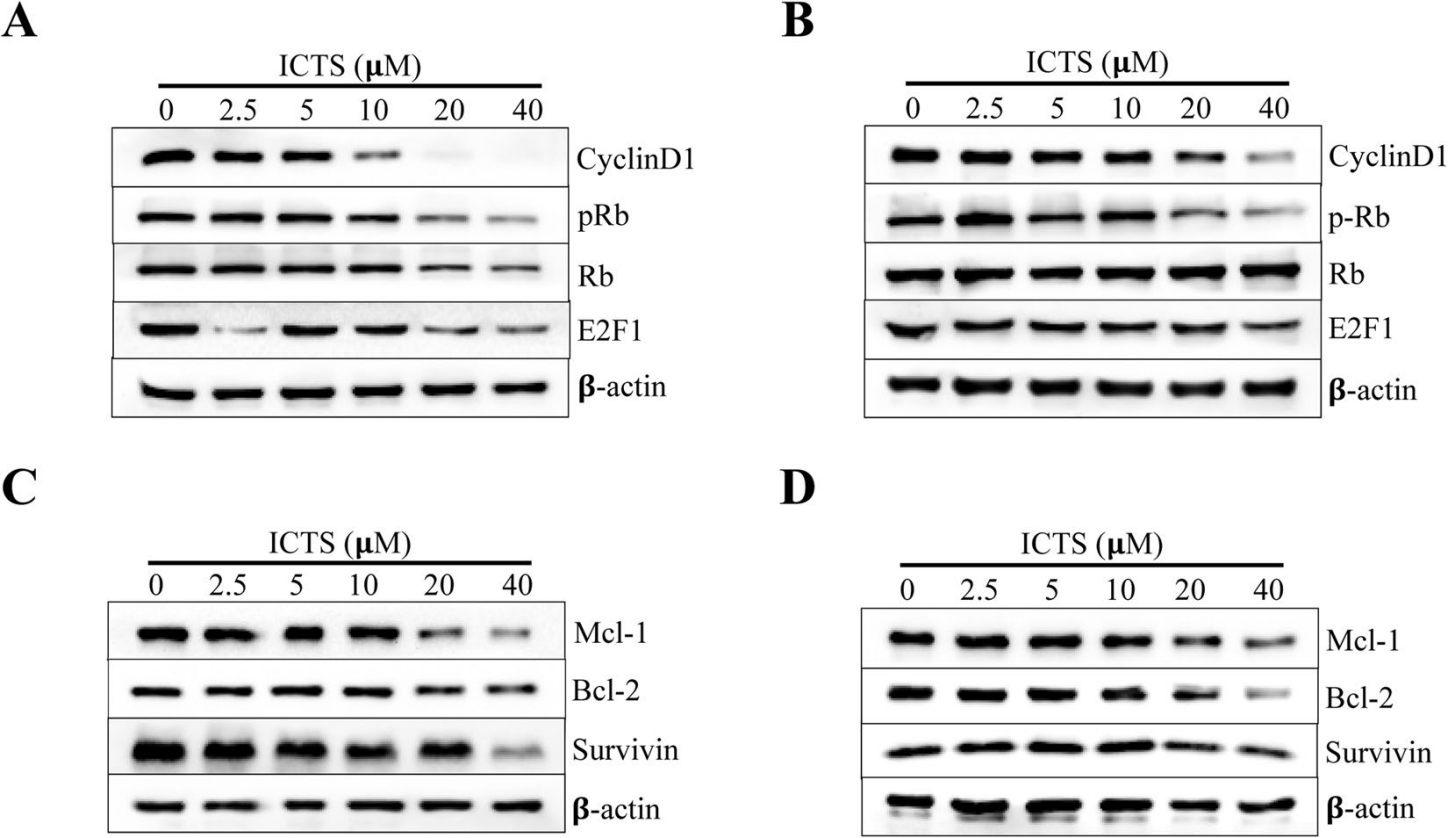
Effect of isocryptotanshinone on expression of cell cycle- and apoptosis-associated proteins in SGC-7901 and MKN-45 cells. SGC-7901 (**A**) and MKN-45 (**B**) cells were serum-starved overnight and treated with the indicated concentration of ICTS and serum for 24 hours, and the expression of Cyclin D1, pRb, Rb, and E2F1 was detected using the Western blot analysis. SGC-7901 (**C**) and MKN-45 (**D**) cells were exposed to the indicated concentration of ICTS for 24 hours, and the expression of Mcl-1, Bcl-2, and Survivin was assessed using the Western blot assay. β-actin was used as the loading control. ICTS, isocryptotanshinone.

To further identify the molecular mechanism of GC cell growth inhibition induced by ICTS, we investigated the expression of cell cycle- and apoptosis-associated protein markers after treatment with ICTS. As shown in Fig. 4A, in SGC-7901 cells, treatment with a concentration of 20 or 40 *μ*M ICTS significantly downregulated the phosphorylation of Rb at Ser-807/811, and the expression of Cyclin D1 and E2F1, which induces the transcription of target genes required for DNA synthesis in the late G1/S phase^29^. Meanwhile, as shown in Fig. 4B, after treatment with a high concentration of ICTS (20 and 40 *μ*M) for 24 hours, the expression levels of Mcl-1, Bcl-2, and Survivin were also decreased significantly in SGC-7901 cells. The decrement of cell cycle- and apoptosis-associated proteins was consistent with the inhibition of cell proliferation. Similarly, as shown in Fig. 4C and D, the same changes of protein markers induced by ICTS in MKN-45 cells were also observed. These results further confirmed the cell cycle arrest and apoptosis induced by ICTS in the GC cells.

### ICTS suppressed phosphorylation of STAT3

**Figure 5:**
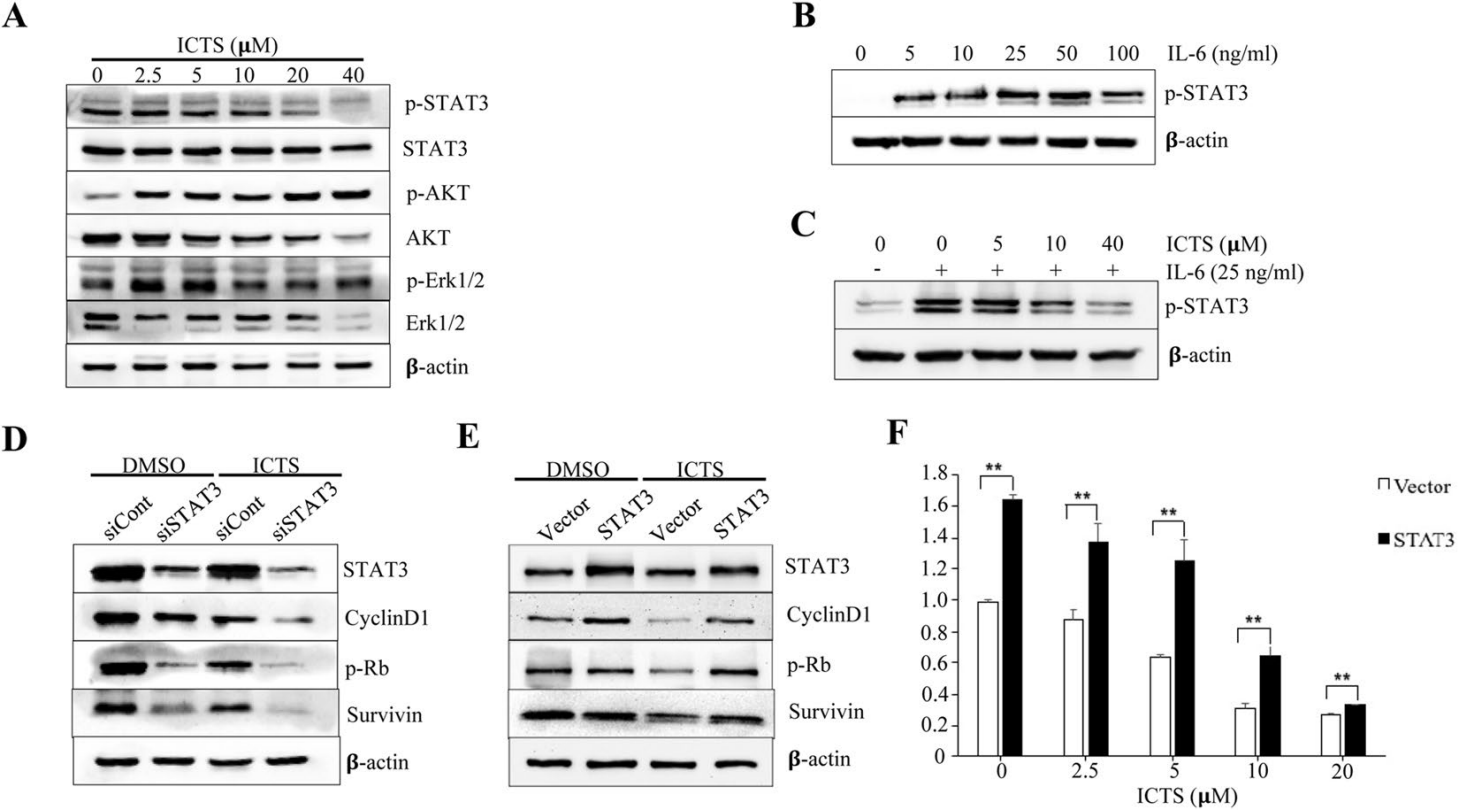
Effect of isocryptotanshinone on signaling pathways in SGC-7901 cells. (**A**) SGC-7901 cells were treated with the indicated concentration of ICTS for 24 hours, and the expression levels of p-STAT3, STAT3, p-Akt, Akt, p-Erk1/2, and Erk1/2 were detected by western blot analysis. (**B**) SGC-7901 cells were deprived of IL-6 overnight and stimulated with the indicated concentration of IL-6 for 30 min. (**C**) SGC-7901 cells were deprived of IL-6 overnight. The cells were treated with ICTS (0, 5, 10, and 40 *μ*M) for 6 hours, and then stimulated with IL-6 (25 ng/ml) for 30 min. The level of p-STAT3 was determined by western blot assay. (**D**) SGC-7901 cells were transfected with siRNA against STAT3 (siSTAT3) or negative control siRNA (siCont) for 48 hours. After treatment with 40 *μ*M ICTS for 24 hours, the expression levels of STAT3, Cyclin D1, p-Rb, and Survivin were assessed using western blot assay. (**E** and **F**) SGC-7901 cells were transfected with vector or STAT3 plasmids for 24 hours and then exposed to 10 *μ*M ICTS for 24 hours. The protein expression and cell growth were detected. β-actin was used as the loading control. **P < 0.01 versus the vector group. ICTS, isocryptotanshinone.

As Jak2/STAT3, MAPK, and PI3K/Akt signaling pathways played pivotal roles in the occurrence and development of GC^30^, we assessed whether ICTS had a regulatory effect on the indicated pathways. As shown in Fig. 5A, ICTS inhibited the phosphorylation of STAT3 at Tyr-705 in a dose-dependent manner and had weak effect on the total protein. It increased the phosphorylation of Akt at Ser-473 and no significant effects were observed on the phosphorylation of Erk1/2 at Thr-202/Tyr-204. However, ICTS, at a higher concentration, decreased the expression levels of Akt and Erk1/2. Interleukin (IL)−6, a pro-tumorigenic cytokine, is associated with poor survival in GC patients and *Helicobacter pylori*-induced STAT3 activation at Tyr-705^31,32^. As shown in Fig. 5B and C, phosphorylation of STAT3 was upregulated remarkably after stimulated by 25 ng/ml IL-6 treatment in SGC-7901 cells, and pre-treatment with ICTS significantly suppressed the IL-6-induced phosphorylation of STAT3. As shown in Fig. 5D, downregulation of STAT3 attenuated the expression of Cyclin D1, p-Rb, and Survivin, which remarkably increased the sensitivity of ICTS in SGC-7901 cells. Overexpression of STAT3 enhanced the growth of the SGC-7901 cells and the expression of Cyclin D1, p-Rb, and Survivin, which restored the cell proliferation and the protein expression suppressed by ICTS (Fig. 5E and F). Together, ICTS inhibited GC cell growth and decreased the expression levels of cell cycle- and apoptosis-associated proteins via inhibiting the STAT3 signaling pathway.

### ICTS inhibited xenograft GC growth in nude mice

**Figure 6:**
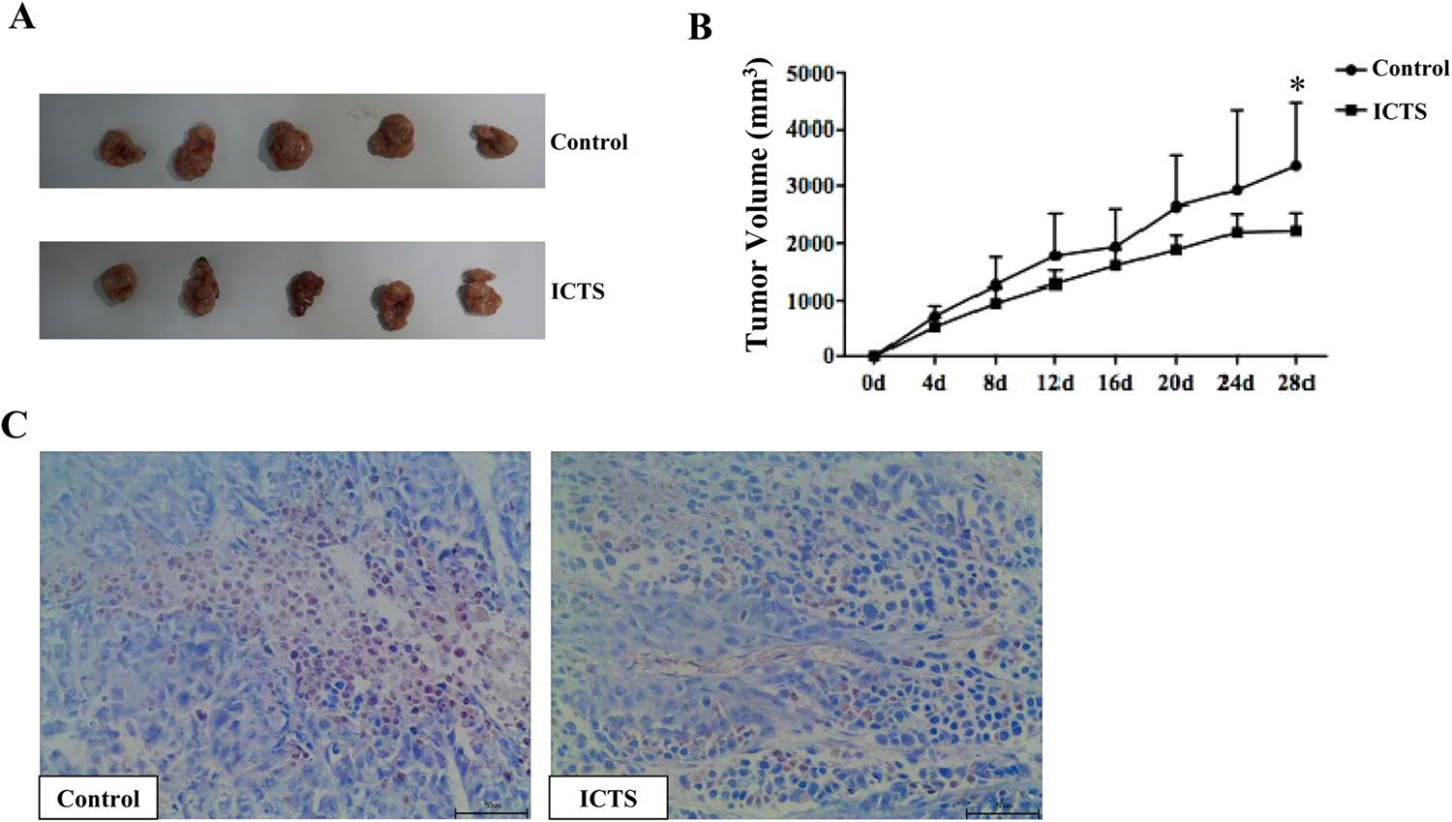
Isocryptotanshinone inhibited xenograft tumor growth in nude mice. The 4-week-old male nude mice were injected subcutaneously with 10^6^ SGC-7901 cells. Two weeks post-xenotransplantation, the mice with tumor volume >200 mm^3^ were intraperitoneally injected with 20 mg/kg ICTS or vehicle every other day for a total of 4 weeks. Tumor size of the nude mice was measured every 4 days. Tumor volume (V) was calculated as: V = π/6 × a × b^2^. (**A**) shows the gross observation of SGC-7901 cell xenograft tumors in nude mice. (**B**) shows the changes of tumor volume which were expressed as mean ± standard error (n = 5, *P < 0.05 for ICTS versus control group). (**C**) indicates the phosphorylation of STAT3 in xenograft tumor detected by immunohistochemistry (high power field, × 400). ICTS, isocryptotanshinone.

To determine the effect of ICTS on xenograft tumor growth *in vivo*, we established the subcutaneous SGC-7901 tumor model. The mice were randomly divided into two groups which received ICTS or vehicle injected intraperitoneally. The results showed a gradual increase in tumor volume in both groups. However, compared with the vehicle group, the tumor volume in the ICTS group increased significantly slower. On the 28^th^ day, the difference of tumor volume between the control and ICTS groups were significant (Fig. 6A and B). Furthermore, phosphorylation of STAT3 in xenograft tumor was identified using immunohistochemistry assay. Results showed that phosphorylation of STAT3 in SGC-7901 tumor was suppressed by ICTS **(**Fig. 6C**)**. Thus, ICTS inhibited SGC-7901 xenograft tumor growth *in vivo*.

## Discussion

In the present study, we found that ICTS significantly inhibited the proliferation of both undifferentiated (MKN-45) and moderately differentiated (SGC-7901) GC cells in a dose- and time-dependent manner, and slowed the xenograft SGC-7901 tumor growth in mice. Cell proliferation is controlled by the activation of the checkpoints during DNA synthesis and chromosome segregation, which protects cells from the attack by genotoxic agents and leads to the inhibition of cyclin-dependent kinases (CDKs) and cell cycle arrest^33^. Three interphase CDKs (CDK2, CDK4, and CDK6), mitotic CDK1, and 4 different classes of cyclins (Cyclin A, Cyclin B, Cyclin D, and Cyclin E) are directly involved in driving the cell cycle and the aberrant expression of the CDK-cyclin complexes resulting from cancer-associated mutations induces unscheduled re-entry into the cell cycle or proliferation^34^. For instance, Cyclin D1, Cyclin D2, and Cyclin D3 bind and activate CDK4 and CDK6 to phosphorylate the Rb protein, which leads to the subsequent release of E2F1 and the transition from G1 to S phase in the cell cycle^35^. Our present study showed that ICTS induced cell cycle arrest in the G1/G0 phase and inhibited the expression of Cyclin D1, pRb, and E2F1 in GC cells in a dose-dependent manner. As an essential protein in the G1-to-S phase transition, E2F1 binds to its dimerization partner 1 or 2 and induces the transcription of the target genes required for DNA synthesis in S phase, such as Cyclin D1, Cyclin E, CDC2, and CDC25A^36^. Park *et al*.^24^ found that cryptotanshinone diminished the E2F1 transcriptional activity compared with the empty vector. Interestingly, this study showed that 2.5 *μ*M ICTS suppressed the expression of E2F1 significantly compared with the 5 *μ*M group after multiple tests. Therefore, the down-regulation of the cell cycle-associated proteins might result from the inhibition of the transcriptional activity of E2F1. Cryptotanshinone-induced cell cycle arrest in the G1/G0 or G2/M phase was dependent on the cell line type, and the effect of ICTS on cell cycle arrest might also be not the same in other cancer cells, which is deserved to be further explored^37^.

To investigate the effect of ICTS on apoptosis of GC cells, the percentage of apoptotic cells was analyzed using flow cytometry and the anti-apoptotic proteins were detected by the Western blot analysis. The results indicated that ICTS arrested GC cell cycle in the G1/G0 phase and inhibited the expression of Mcl-1, Bcl-2, and Survivin, which was in accordance with the experimental results in A549 and MCF-7 cells^26,27^. Mcl-1 and Bcl-2, the anti-apoptotic proteins of the Bcl-2 family, regulate programmed cell death via directly inhibiting the pro-apoptotic proteins^38^. Recent studies showed that cryptotanshinone induced apoptosis of pancreatic cancer^39^, prostate cancer^40^, chronic myeloid leukemia^41^, multiple myeloma^42^, glioma^43^, and lung cancer^44^ cells by suppressing the anti-apoptotic proteins. Chen *et al*.^45^ found that the cryptotanshinone-induced caspase-dependent cell death inhibited the expression of anti-apoptotic (Bcl-2 and Mcl-1) and survival proteins (Survivin) by activating the Jun N-terminal kinase (JNK) pathway in cancer cell lines. The Bcl-2 family proteins were proved to be down-regulated by nature compounds, which were already used in clinical trials^46^. Small-molecule suppressors of Survivin (*e.g*., YM155, Tetra-O-methyl nordihy-droguaiaretic acid, and LY2181308) were reported to show anti-cancer activities both *in vitro* and *in vivo*, and some of them have been applied in clinical anti-cancer therapy^47–51^. Our *in vivo* investigation further suggested that ICTS might be a nature compound which could be potentially applied in clinical GC treatment, and further investigations are warranted in this regard.

The persistent activation of STAT3 was found in multiple human cancers^52^, and plays a prominent role in mediating drug resistance during chemotherapy and targeted cancer therapies^53–55^. Researches have shown that activated STAT3 promoted the proliferation and invasion of GC cells *in vitro*^56,57^. Additionally, STAT3, as a prognostic marker, was associated with a poor survival in GC^58^. Serum IL-6 expression was an independent indictor for survival and a high expression level was associated with cancer development and progression in GC^32,59^. In this study, we demonstrated that ICTS attenuated the phosphorylation of STAT3 stimulated by IL-6 in GC cells, which is consistent with the results in A549 cells^26,27^. It has been found that the activation of STAT3 elevated the levels of anti-apoptotic (Mcl-1 and Survivin) and cell cycle-regulating proteins (Cyclin D1)^60,61^. When STAT3 was down-regulated by siRNA transfection, the expression of CylinD1 and Survivin was also significantly decreased (Fig. 5B**)**. Meanwhile, upregulation of STAT3 attenuated the inhibition of Cyclin D1, p-Rb, and Survivin induced by ICTS. Whether ICTS, as a STAT3 suppressor, could obviously enhance the sensitivity of chemotherapy in GC needs further research.

Several issues are noteworthy in this study. Firstly, the efficacy of the investigated compound is relatively low as reflected by the proliferation assay. Nevertheless, based on our data and one publication^62^, the inhibition potency of ICTS was stronger than cryptotanshinone in SGC-7901 and MKN-45 cells. Different inhibitory effects of ICTS on different gastric cancer cells could be due to their discrepant differentiation potentials and cell types, which would be explained by further related studies. Secondly, after treatment by ICTS at 10 µM, >70% of the growth-inhibited SGC-7901 cells did not undergo apoptosis, indicating that the observed cell death could be largely due to reasons other than apoptosis. We also suggested that cell cycle inhibition would be one of the mechanisms, the others of which warrant further exploration. Notably, p-AKT was increased but total AKT inhibited by ICTS in a concentration-dependent manner (Fig. 5A), and the underlying mechanism calls for further explorations.

In conclusion, the present study provided the first evidence that ICTS inhibited GC cell proliferation by inducing cell cycle arrest and apoptosis through inhibition of the STAT3 signaling.

## Materials and Methods

### Reagents

ICTS was obtained from ChemFaces, and the purity (98%) was verified using high performance liquid chromatography. Cell Counting kit (CCK)−8 and propidium iodide (PI) were obtained from Beyotime, and FITC Annexin V Apoptosis Detection Kit was purchased from BD Biosciences. IL-6 was purchased from PeproTech. Primary antibodies used in Western blot were Cyclin D1, phosphorylated Rb (Ser-807/811), E2F1, Mcl-1, Survivin, Bcl-2, p-STAT3 (Tyr705), STAT3, p-Erk1/2 (Thr202/Tyr204), Erk1/2, p-Akt (Ser473), Akt (Cell Signaling Technology), β-actin (Bioworld), and Rb (Abconal), and secondary antibodies were anti-rabbit/mouse IgG (ZSGB-Bio).

### Cell culture

Human GC cell line SGC-7901 was a kind gift from Dr. Ping Wu (Anhui Medical University), and MKN-45 was purchased from Cell Bank of Chinese Academy of Sciences. They were maintained in DMEM (SGC-7901) and RPMI 1640 (MKN-45), respectively, supplemented with 10% (v/v) fetal bovine serum and 1% (v/v) penicillin-streptomycin in a 37 °C incubator with 5% CO_2_.

### Cell proliferation assay

The effect of isocryptotanshinone on cell proliferation was determined by CCK-8 assay. Briefly, cells were cultured in 96-well plates at a density of 2 × 10^3^ cells per well and attached overnight, and they were then treated with isocryptotanshinone of different concentrations or DMSO (Beyotime) as vehicle and incubated for appropriate time. CCK-8 solution was added followed by incubation for 1 to 3 hours at 37 °C and with 5% CO_2_, and the absorbance per well was measured at 450 nm wavelength using a universal microplate reader (Bio-tek). Results were shown as the relative ratio compared with the control group which was set as 1.

### Cell cycle analysis

Cells were seed in 6-well plates at a density of 1 × 10^5^ cell per well and serum-starved overnight. After treated with 10% serum and isocryptotanshinone (0–40 *μ*M) or DMSO for 24 hours, cells were harvested and washed with cold phosphate buffer saline (PBS) twice and then fixed with 70% cold ethanol for 24 hours. Fixed cells were washed again and stained with 500 μL PI solution. After incubation in 37 °C away from light for 30 min. The stained cells were analyzed by FACScan flow cytometry (Becton-Dickinson Biosciences) and CellQuest software. Percentages of cells in sub-G1, G1/G0, S, G2/M phase of cell cycle were determined by using Flow Jo software (v. 7.6.1).

### Cell apoptosis detection

Cells were seeded into 6-well plates at a density of 1 × 10^5^ cells per well and allowed to grow overnight. After treated with isocryptotanshinone (2.5–40 *μ*M) or DMSO for 24 hours, cells were harvested and washed twice using cold PBS, and re-suspended in Annexin-binding buffer containing PI and annexin-v in dark at room temperature for 15 minutes. The stained cells were subjected to FACScan flow cytometry (Becton-Dickson Biosciences) using the CellQuest software, and the percentage of apoptotic cells was analyzed using the Flow Jo software.

### siRNA/plasmid transfection

The double-strand siRNA was synthesized by Genepharma (Shanghai). The sequence of STAT3-siRNA was as follows: sense, 5′-UGUUCUCUGAGACCCAUGATT-3′; antisense, 5′-UCAUGGGUCUCAGAGAACATT-3′^63^. The sequence of the negative control was as follows: sense, 5′-UUCUCCGAACGUGUCACGUTT-3′; antisense, 5′-ACGUGACACGUUCGGAGAATT-3′. The STAT3 and empty vector plasmids were purchased from Quanayang (Shanghai) and verified with sequencing. SGC-7901 cells were seeded into 6-well plates with a final confluence of 50–60% and allowed to grow overnight. Cells were transfected with siRNAs or plasmids using Lipofectamine 2000 (Invitrogen) according to the manufacturer’s instructions. After treated with isocryptotanshinone or DMSO for 24 hours, cells were lysed and then analyzed by Western blotting.

### Western Blot analysis

After treatment with isocryptotanshinone or DMSO, cells were lysed in RIPA lysis buffer containing 1 mM PMSF (Beyotime). The concentration of protein samples was detected using the BCA Protein assay kit (Beyotime). About 30 micrograms of the total protein were separated using 10% SDS-PAGE and transferred to a PVDF membrane. After blocked in TBS-T (20 mM Tris-HCl (pH 7.4), 150 mM NaCl, and 0.1% Tween) containing 5% non-fat milk for 90 minutes, the membranes were incubated with primary antibodies at 4 °C overnight. The membranes were then washed with TBS-T, and exposed to HRP-conjugated goat-anti-rabbit/mouse secondary antibodies at room temperature for 60 minutes. Blots were detected by chemiluminescence using Super Signal West Femo (Thermo Scientific) and bands were captured using a digital imaging system (Tanon).

### Animal experiment

Ten 4-week-old male BALB/c nude mice (weight, 20 ± 4 g) were obtained from Beijing Vital River Laboratory Animal Technology Co. Ltd. All mice were maintained under specific pathogen free conditions at 22 °C with 55% humidity and a 12-h light/12-h dark cycle. 10^6^ SGC-7901 cells were subcutaneously injected into the upper flank region of the nude mice. After 2 weeks, all mice were divided into two (ICTS and control) groups which were intraperitoneally injected with 20 mg/kg ICTS or vehicle every other day for a total of 4 weeks. Tumor sizes of nude mice were measured every 4 days. Tumor volumes (V) were calculated following V = π/6 × a × b^2^. At the end of the experiments, mice were subsequently sacrificed by cervical dislocation after anesthesia.

Then the SGC-7901 tumors were harvested and fixed in 4% poly-formal-dehyde for 2 days. We detected the phosphorylation of STAT3 in tumors by immunohistochemistry staining as previously described^15,64^. The primary antibody replaced by PBS was regarded as the negative control and the known positive tissue section staining was considered as the positive control.

### Statistical analysis

The quantitative data obtained from at least triplicate wells were presented as mean ± SE. The non-linear regression analysis was performed using the GraphPad Prism software to calculate the IC_50_. The qualitative differences between 2 groups were analyzed using the Student’s *t* test. *P* values of <0.05 and <0.01 were considered statistically significant and very significant, respectively.

### Ethics statement

The *in vivo* part of the current study was performed according to the National Institutes of Health Guidelines for the Care and Use of Laboratory Animals and approved by the Animal Care and Use Committee of Anhui Medical University.
